# Monitoring of malaria vectors at the China-Myanmar border while approaching malaria elimination

**DOI:** 10.1186/s13071-018-3073-4

**Published:** 2018-09-15

**Authors:** Shao-sen Zhang, Shui-sen Zhou, Zheng-bin Zhou, Tian-mu Chen, Xue-zhong Wang, Wen-qi Shi, Wei-kang Jiang, Ju-lin Li, Xiao-nong Zhou, Roger Frutos, Sylvie Manguin, Aneta Afelt

**Affiliations:** 1 National Institute of Parasitic Diseases, Chinese Center for Disease Control and Prevention, Shanghai, 200025 China; 2 Key Laboratory of Parasite and Vector Biology, Ministry of Health, Shanghai, 200025 China; 3 National Center for International Research on Tropical Diseases, Ministry of Science and Technology, Shanghai, 200025 China; 4 WHO Collaborating Center for Tropical Diseases, Shanghai, 200025 China; 5 IES, Université Montpellier, CNRS, 34059 Montpellier Cedex 5, France; 6 Cirad, UMR 17, Intertryp, Campus international de Baillarguet, 34398 Montpellier Cedex 5, France; 7 HydroSciences Montpellier (HSM), Institut de Recherche pour le Développement (IRD), CNRS, Université Montpellier, 34093 Montpellier, France; 8 Yunnan Institute of Parasitic Diseases, Pu’er Yunnan, 665000 China; 9 Jiangsu Institute of Parasitic Diseases, Wuxi, 214064 Jiangsu Province China; 10 Interdisciplinary Center for Mathematical and Computational Modelling, University of Warsaw, Tyniecka 15/17, 02-630 Warsaw, Poland

**Keywords:** Malaria vector, China-Myanmar border, Malaria elimination, Ecological traits, Receptivity

## Abstract

**Background:**

Tengchong County was one of the counties located at the China-Myanmar border with high malaria incidence in the previous decades. As the pilot county for malaria elimination at the border area, Tengchong County is aiming to be the first county to achieve malaria elimination goal. A cross-sectional entomological survey was carried out to evaluate the feasibility of elimination approach and assess the receptivity of malaria reintroduction.

**Methods:**

Light traps associated with live baits were used to investigate the abundance of adult mosquitoes in nine villages in Tengchong County. Light traps were set to collect adult mosquitoes in both human houses and cowsheds from dusk till dawn in each site.

**Results:**

A total of 4948 adult *Anopheles* mosquitoes were collected from May to December in two villages. Of the mosquitoes were captured, 24.2% were in human houses and 75.8% in cowsheds. The peak of abundance occurred in July for *An. sinensis* and in September-October for *An. minimus* (*s.l.*) Ten *Anopheles* species were collected, the most prevalent being *An. sinensis* (50.3%), *An. peditaeniatus* (31.6%) and *An. minimus* (*s.l.*) (15.8%), contributing to 97.6% of the sample. Potential breeding sites were also investigated and a total of 407 larvae were collected, with *An. sinensis* (50.1%) and *An. minimus* (*s.l.*) (46.2%) as predominant species. Ponds and rice fields were the two preferred breeding sites for *Anopheles* mosquitoes; however, the difference between the number of adults and larvae captured suggest other breeding sites might exist. Both *An. sinensis* and *An. minimus* (*s.l.*) were found zoophilic with human blood index as 0.21 and 0.26, respectively. No *Plasmodium* positive *Anopheles* specimens were found by PCR among 4,000 trapped mosquitoes.

**Conclusions:**

Although no indigenous malaria cases have been reported in Tengchong County since 2013, there is still a risk from the presence of vectors in the context of human population movements from neighboring malaria endemic areas. The presence of *An. sinensis*, associated to rice fields, is particularly worrying. Sustained entomological surveillance is strongly suggested even after malaria elimination certification.

## Introduction

Malaria is the deadliest vector-borne disease in tropical and subtropical areas, with a number of confirmed cases estimated at 216 million with 445,000 deaths in 2016 [1]. Out of 91 countries and territories with malaria transmission in 2016, 44 reported less than 10,000 cases and 21 are approaching malaria elimination, including China. Significant progress on malaria control has been made during the past decades and China is now aiming to achieve malaria elimination by 2020 [2, 3]. However, malaria control in international border areas is considered a challenge, especially at the China-Myanmar border in Yunnan Province [4–6]. Most of indigenous malaria cases (up to 90%) and malaria cases imported from Southeast Asia within China have been reported along this border since 2013 [7–11].

**Fig. 1:**
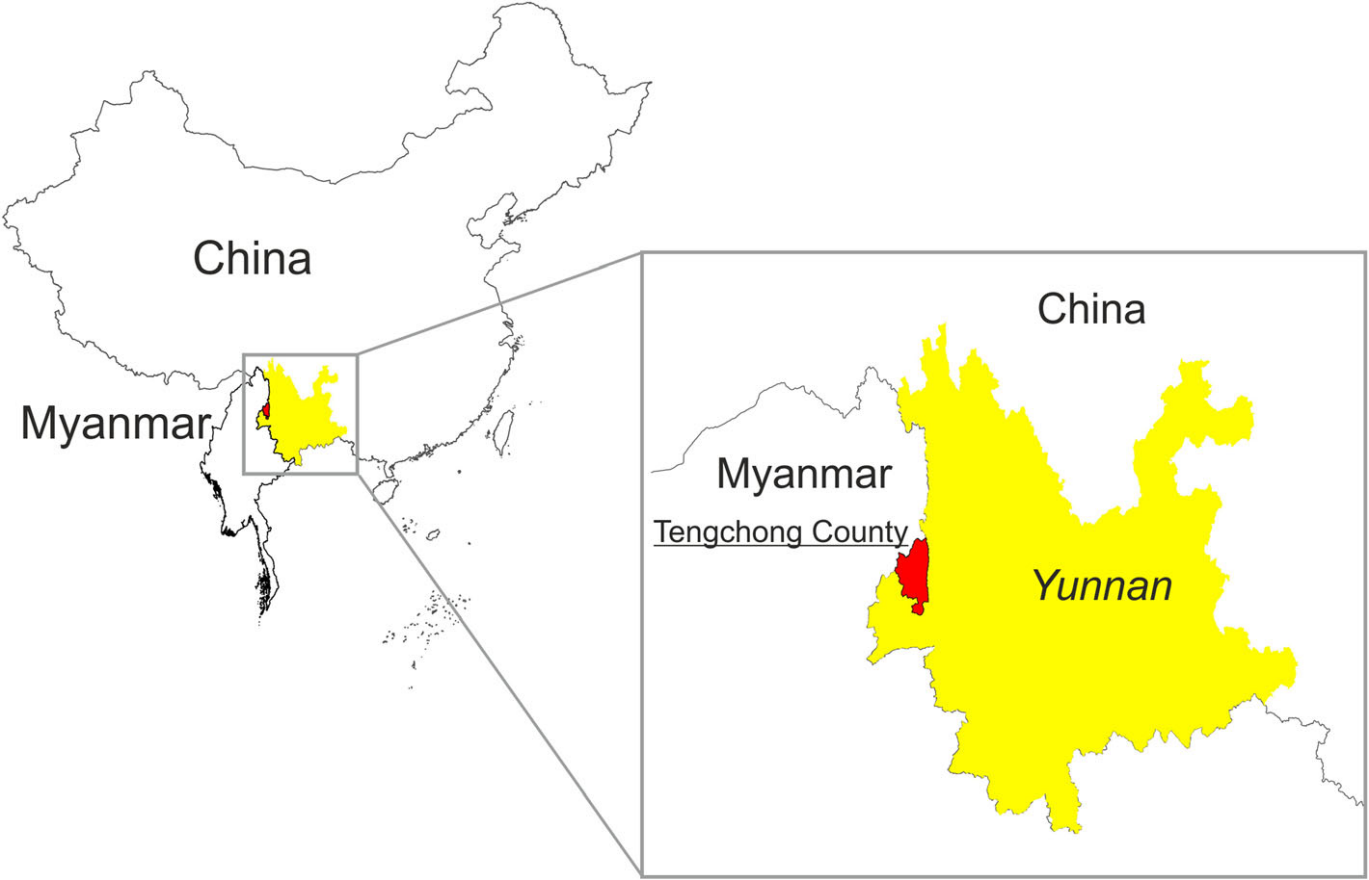
Location of Tengchong County in the China-Myanmar border area

Tengchong County (TCC) is located in the southwest of Yunnan Province at the China-Myanmar border (Fig. 1). Because of the diversity of malaria vectors and large population movements across the border, the number of malaria cases reported in TCC was the highest for the whole country in previous years [6, 12]. Hence, TCC was designated in 2012 as the pilot county for malaria elimination at border areas with the objective of being the first border county to achieve malaria elimination. This status was officially granted in 2015 and no locally transmitted cases were observed since then. However, imported cases from neighboring Myanmar were recorded [8, 13, 14]. To investigate the feasibility of malaria elimination and assess the risk of reintroduction of malaria in TCC, a series of studies and analyses on epidemiology were carried out and published recently [8, 14]. These studies emphasized the risk of reintroduction of malaria in TCC due to population movement across the border into putative receptive areas. However, malaria vectors are the key drivers for malaria transmission and reintroduction [15–17]. We therefore investigated the presence of primary and secondary vectors in TCC through a cross-sectional survey.

## Methods

### Study sites

**Fig. 2:**
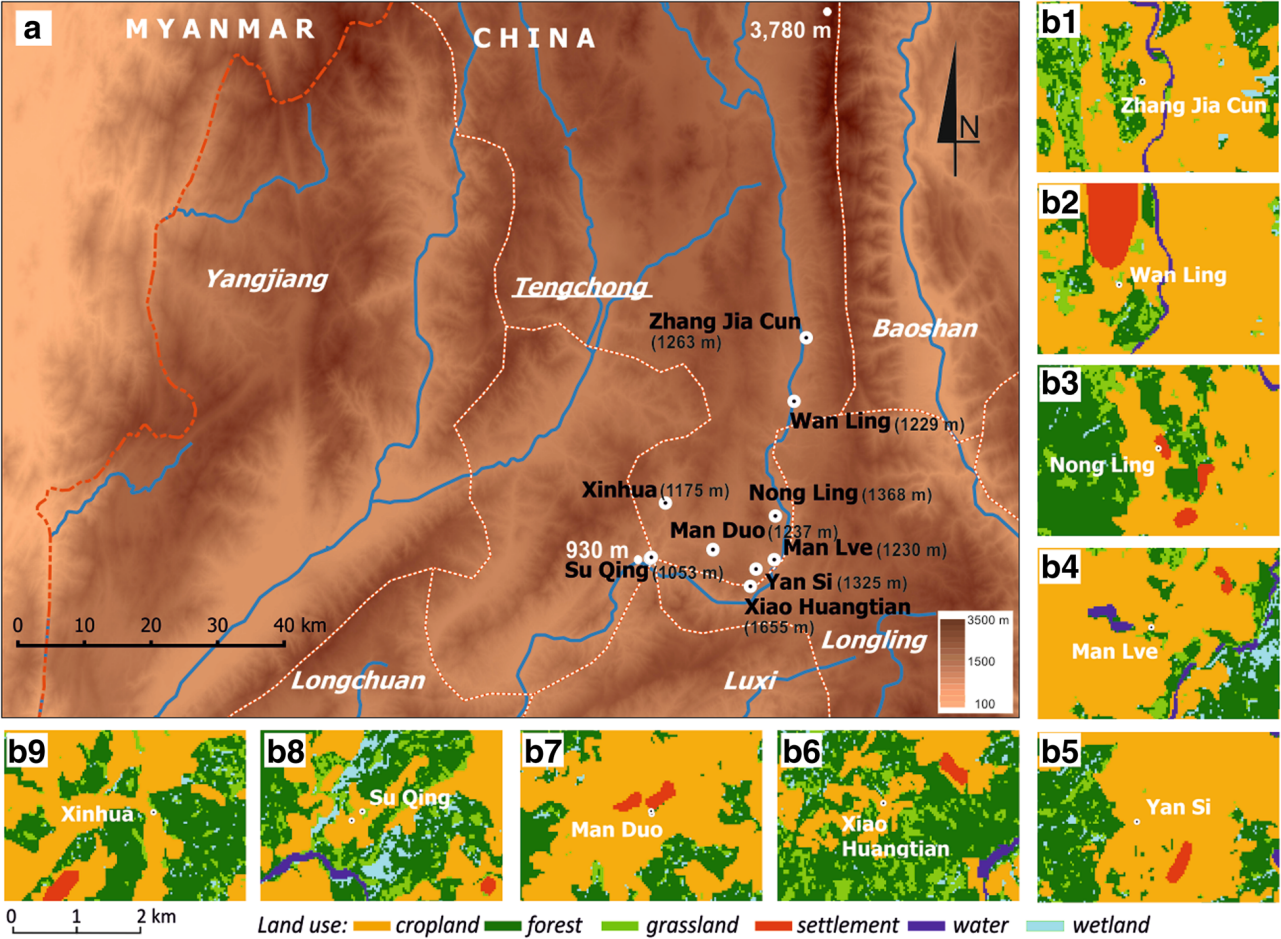
Map of the of the study site. **a** Location of study sites in the Tengchong County. **b** 1–9. Land use around each village

TCC covers an area of 5693 km^2^ with a population of 6.68 million inhabitants and an international borderline with Myanmar of 148 km (98°05'E–98°45'E, 24°38'N–25°52'N). Mountains cover 84% of the territory with a maximum elevation of 3780 m (Fig. 2a). The altitude decreases from northwest to southeast with the lowest point at 930 m (Fig. 2a). The annual average rainfall is 1531 mm and the relative humidity is 77%, displaying the typical characteristics of a subtropical monsoon climate. The annual average temperature is 15 °C, decreasing to 0 °C during winter (January). The rainy season lasts from June to September. Nine villages located predominantly along the main regional river and with an elevation between 1032–1655 m were investigated (Fig. 2a, Table 1). These villages were chosen because they displayed the highest number of imported cases and the highest population movements across the international border with Myanmar, which is located 70 km away [5, 6, 8, 14, 18]. Land use and land cover (LULC) in TCC displayed two main features: (i) either predominantly cropland landscape; or (ii) predominantly forest mixed with grasslands (Fig. 2b). Study sites were selected also in relation to urbanized area, either: (i) at the edge of village area; (ii) close to village; or (iii) away from urbanized area (Table 1, Fig. 2b). Two sites were located close to the dense forest, Yan Si and Xinhua (Table 1, Fig. 2b5, b9), whereas five sites were next to fragmented forest, mixed with grasslands: Zhang Jia Cun; Nong Ling; Man Lve; Xiao Huangtian; and Su Qing (Table 1, Fig. 2b1, b3, b4, b6, b8). The last two sites, i.e. Wan Ling and Man Duo, were located in cropland areas nearby urbanized zones (Table 1, Fig. 2b2, b7).

**Table 1:** Sampling sites with village names, location, altitude, type of landscape and forest, and distance to urbanized area

Village	Township	Latitude (°N)	Longitude (°E)	Altitude (m)	Landscape	Type of forest	Urbanized area^a^
Xiao Huangtian	Tuan Tian	24.648424	98.611341	1655	Forest, grassland	Fragmented	Away
Yan Si	Tuan Tian	24.668723	98.619704	1325	Cropland	Dense	Away
Man Lve	Tuan Tian	24.679774	98.646722	1230	Cropland	Fragmented	Away
Nong Ling	Tuan Tian	24.730656	98.648392	1368	Forest, grassland	Fragmented	Edge
Wan Ling	Wu He	24.865358	98.676102	1229	Cropland	Away from fragm. forest	Close
Zhang Jia Cun	Mang Bang	24.939534	98.694098	1270	Cropland	Fragmented	Away
Man Duo	Pu Chuan	24.691051	98.555936	1232	Cropland	Away from fragm. forest	Edge
Su Qing	Xin Hua	24.681433	98.462895	1032	Forest, grassland	Fragmented	Away
Xinhua	Xin Hua	24.745937	98.485421	1175	Forest, grassland	Dense	Away

^a^Urbanized area located (i) away, (ii) at the edge or (iii) close to villages

*Abbreviation*: fragm., fragmented forest

### Mosquito collection and species identification

#### Adult mosquitoes

The collection of adult mosquitoes was conducted from May to December 2015 in two villages, Man Lve and Nong Ling, which are located 3 km apart and characterized by a difference in elevation and surrounded by forest, croplands and grasslands (Table 1, Fig. 2). Light traps were set up to collect adult mosquitoes in both human houses and cowsheds from sunset to sunrise (times varied depending on the season). Humans under bed nets and cattle were used as biological baits for mosquito collection using light traps in houses and cowsheds, respectively. These human and animal-occupied structures were selected in each village at differing distance from farmlands (farmlands are always located around the village), i.e. close, mid-distance and far from the farmland. Sampling was conducted for two nights every month, one night at the beginning of the month and the other at the end. The same sampling method was implemented in an additional sampling campaign to investigate the *Anopheles* diversity in October 2015 over seven additional villages bringing the overall sampling area to a total of nine villages (Table 1, Fig. 2). The same sampling effort was implemented and the same number of sampling sites was considered in all locations. Trapped mosquitoes were killed by chloroform, counted and identified according to morphological criteria [19].

#### Mosquito larvae

All potential *Anopheles* breeding sites (stream, rice field, small pool, canal, ditch, etc.) around the selected villages were investigated for larvae. The hand dipper sampling method was used to collect larvae (500 ml per dip, 10 dips for each waterbody) [20]. The morphological identification of specimens was only conducted for fourth-instar larvae under light microscope. Larvae under the fourth-instar were only counted but not identified. Pupae were kept until adult emergence in order to conduct morphological and molecular identifications. Both adult and larval specimens were preserved in ethanol for further PCR analysis. A series of multiplex PCR assays based on rDNA internal transcribed spacer 2 (ITS2) and D3 domain of *28S* rDNA sequences were run to identify the sibling species of the *An. minimus*, *An. culicifacies* and *An. fluviatilis* complexes and *An. maculatus* group [21–24].

### Entomological data

Monthly abundance data of each *Anopheles* species were aggregated to analyze seasonal fluctuations. The resting behavior and breeding preference of adult mosquitoes were explored by analyzing the adult and larval composition in each study place. The adult population density for each *Anopheles* species was calculated as the number of females per trap per night (f/t/n). The overall (pooled) *Anopheles* density was calculated by summing captured individuals of all *Anopheles* species. Species richness was measured by the number of species and the indices described below. Generally, species diversity is an indicator of the wellbeing of an ecosystem [25]. Simpson’s diversity index (D) is often used to quantify the biodiversity of an ecosystem. It takes into account the number of species present, as well as the abundance of each species. The value of this index ranges between 0 and 1, 0 representing the absence of diversity and 1 representing infinite diversity. Shannon-Wiener’s index (H) takes into account individuals of each species to assess the species richness. The larger the value of the H index, the higher the diversity. The Evenness index (E) represents the equitability of populations [26]. The larger the value of the E index, the higher the equitability.

The indices were calculated as follows:

Simpson’s diversity index: $\mathrm{D}=1-{\sum}_{i=1}^N{p}_i^2$

Shannon-Wiener’s index: H =  −∑ *pi*×ln *pi*

where *pi* is the fraction of a species which belongs to the i-th species and N is the number of species (*pi* = *N*_*i*_/*N*).

Evenness index: E = *H*/ ln *S*

where *H* is the Shannon-Wiener’s diversity index and *S* is the total number of species observed in a given place.

### Detection of *Plasmodium* spp. in mosquitoes and blood source identification

Captured mosquitoes were dissected into different segments under light microscope. The head and thorax were separated for *Plasmodium* spp. test while the abdomen was used for blood-meal identification. DNA extraction was conducted with QIAamp DNA Mini Kit (Qiagen GmbH, Hilden, Germany) according to the supplier. PCR tests for blood source identification were conducted as previously described [27–29]. Primers are presented in Table 2.

**Table 2:** Primers for *Plasmodium* spp. and blood meal identification

No.	Primer name	Sequence (5'-3')	Product size (bp)
Test for blood meal identification			
1	Human blood	GGCTTACTTCTCTTCATTCTCTCCT	334
2	Pig blood	CCTCGCAGCCGTACATCTC	453
3	Cow blood	CATCGGCACAAATTTAGTCG	561
4	Dog blood	GGAATTGTACTATTATTCGCAACCAT	680
5	UNREV	GGTTGTCCTCCAATTCATGTTA	–
Test for *Plasmodium* spp.			
1	Pf1	CCTGCATTAACATCATTATATGGTACATCT	273
2	Pf2	GATTAACATTCTTGATGAAGTAATGATAATACCTT	
3	Pv1	AAGTGTTGTATGGGCTCATCATATG	290
4	Pv2	CAAAATGGAAATGAGCGATTACAT	

*Abbreviation*: UNREV, Universal reversal primer

### Assessment of the parous rate

**Fig. 3:**
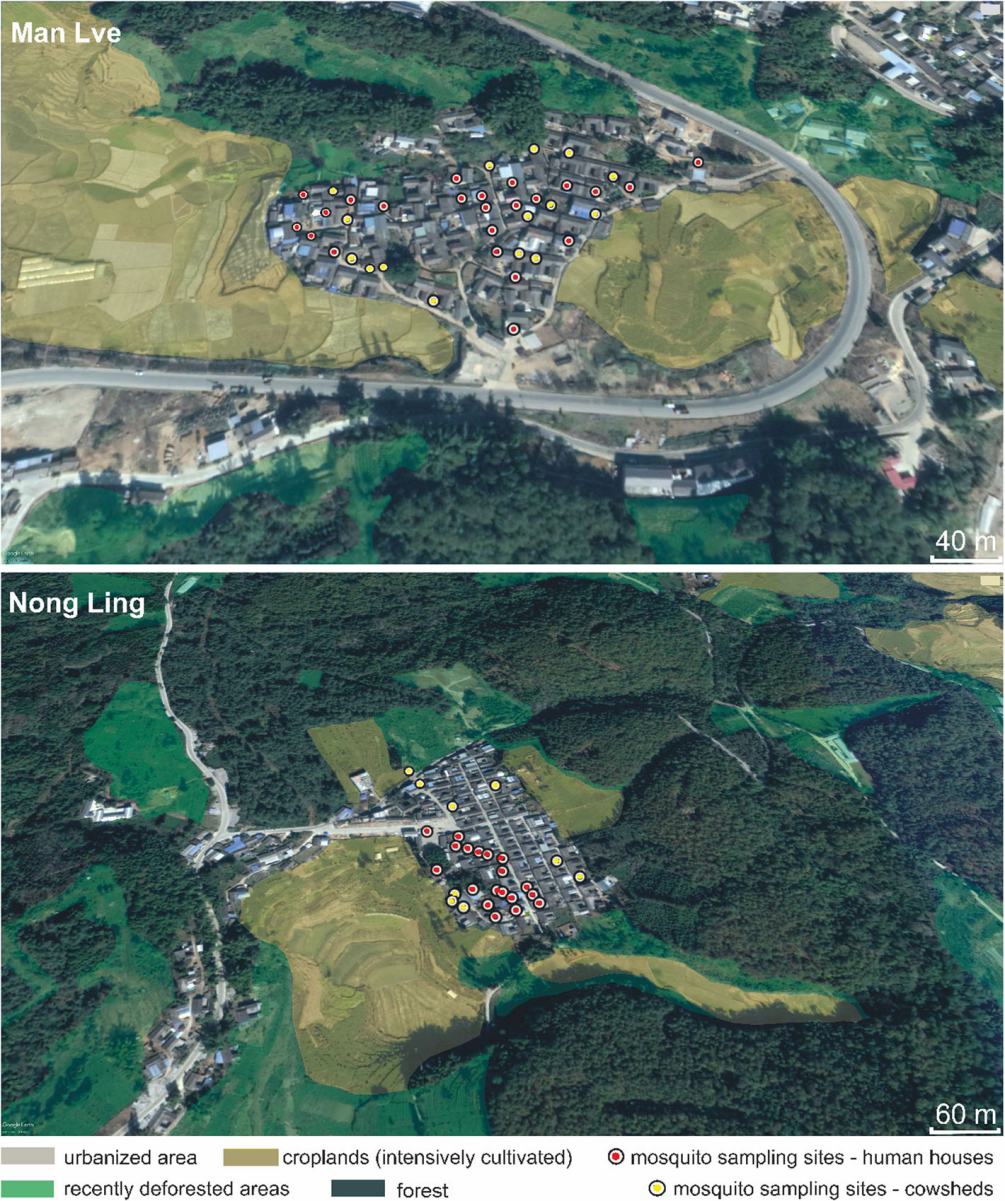
Aerial view of Man Lve (top) and Nong Ling (bottom) with land use and locations of the sampling sites

Mosquitoes were collected by six collectors using an aspirator in different locations, including human houses and cowsheds (Fig. 3). The collected mosquitoes were transported to the laboratory, where they were killed by chloroform and dissected with minute dissection needles for ovarian examination. Ovaries were separated from the other internal organs (including the Malpighian tubules and stomach) and teased apart on slides with deionized water. The slides were checked under light microscope at 10×–40× magnification to confirm whether the mosquitoes were parous or nulliparous.

### Geographical data

Administrative spatial data were obtained from the GADM database of Global Administrative Areas (http://www.gadm.org). The relief model was prepared using SRTM 90m digital elevation data v4.1 [30]. Land cover data were obtained from the GlobeLand30 service operated by the National Geomatics Center of China [31]. Data were initially produced in 2010 and updated in 2014. Images used for GlobeLand30 (GLC30) classification were multispectral images with a 30-meter resolution. Six classes of land cover were displayed: crop land; forest; grassland; wetland; water bodies; and human settlements. Climate description was made using global climate data (Tutiempo Network). Data mapping was performed with Quantum GIS, version 2.8.2.

## Results

### Species richness and diversity

The diversity of species measured by the Simpson’s diversity index (D) and the Shannon-Wiener’s index (H), both for human houses and cowsheds, was at the highest in Man Lve and Nong Ling at the beginning of the fall, i.e. September-October, although the trend was already visible in August (Table 3). The diversity, both in terms of number of species and number of individuals per species, was at the highest during September and October and similar for both locations although indices were displaying some differing trends. Diversity for both species and number of individuals was slightly higher in cowsheds than human houses in Man Lve whereas it was higher in human houses than cowsheds in Nong Ling (Table 3). Evenness followed a similar trend, indicating thus equitability of populations along with the increasing the number of species.

**Table 3:** *Anopheles* species richness and diversity per month, location and village

Month	Species richness^a^		Simpson’s diversity index (D)		Shannon-Wiener’s index (H)		Evenness index (E)	
	Human house	Cowshed	Human house	Cowshed	Human house	Cowshed	Human house	Cowshed
Man Lve								
May	2	3	0.43	0.52	0.52	0.79	0.90	0.72
June	3	4	0.18	0.46	0.46	0.83	0.35	0.60
July	6	4	0.40	0.52	0.52	0.92	0.40	0.67
August	4	6	0.56	0.60	0.60	1.09	0.73	0.61
September	4	5	0.54	0.68	0.68	1.18	0.70	0.73
October	3	4	0.36	0.62	0.62	1.07	0.57	0.77
November	2	4	0.50	0.42	0.42	0.84	1.00	0.60
December	1	1	0	0	0	0	0	–
Nongling								
May	1	1	0	0	0	0	–	–
June	1	3	0	0.12	0	0.28	–	0.25
July	5	4	0.29	0.37	0.59	0.67	0.37	0.49
August	5	3	0.51	0.48	0.90	0.70	0.56	0.64
September	4	3	0.66	0.53	1.13	0.89	0.82	0.81
October	4	5	0.66	0.52	1.15	0.95	0.83	0.59
November	0	0	–	–	–	–	–	–
December	0	0	–	–	–	–	–	–
Cumulative data								
May	2	3	0.35	0.51	0.54	0.77	0.77	0.70
June	3	4	0.13	0.40	0.30	0.75	0.27	0.54
July	6	4	0.36	0.48	0.69	0.85	0.39	0.61
August	6	6	0.53	0.57	1.00	0.98	0.56	0.54
September	5	5	0.64	0.65	1.13	1.10	0.70	0.68
October	4	6	0.63	0.62	1.02	1.08	0.74	0.60
November	2	4	0.50	0.42	0.69	0.84	1.00	0.60
December	1	1	0	0	0	0	0	0

^a^Species richness is defined by the number of species captured

### Variation of adult mosquito incidence over time and space in Man Lve

**Fig. 4:**
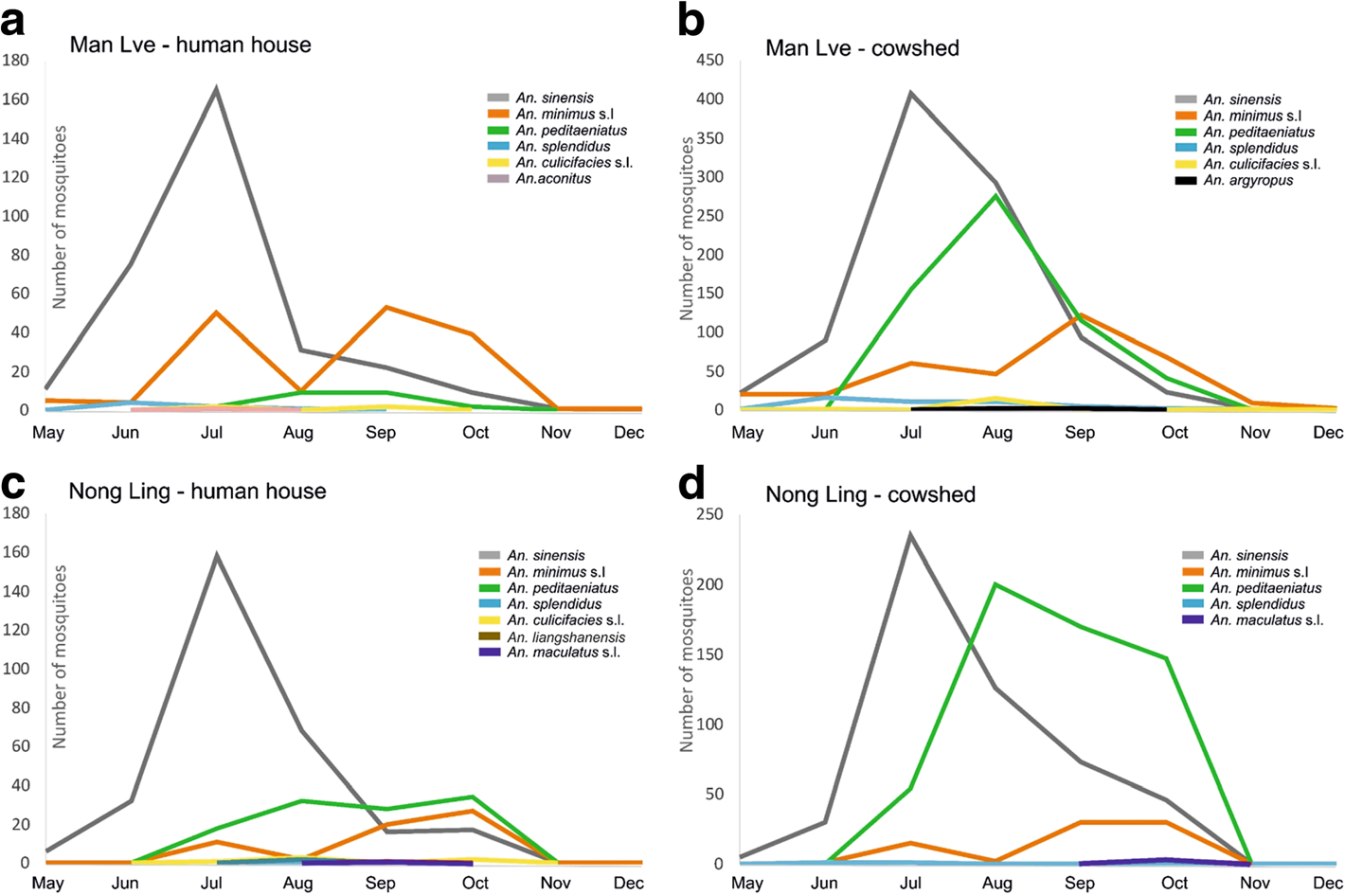
Distribution and seasonal fluctuation of captured *Anopheles* taxa in two study sites, Man Lve (**a**, **b**) and and Nong Ling (**c**, **d**) in human house (**a**, **c**) and cowshed (**b**, **d**)

The collection of adult mosquitoes in the village of Man Lve (Fig. 3) was conducted in two different types of shelters: human houses (Fig. 4a) and cowsheds (Fig. 4b) from May to December 2015. A total of 511 adult mosquitoes were collected inside houses over eight months (Fig. 4a). The most prevalent species were *An. sinensis* (61.4%, 314/511), *An. minimus* (*s.l.*) (31.9%, 163/511) and to a lower extent *An. peditaeniatus* (4.3%, 22/511). Three other species were collected, i.e. *An. splendidus* (1.4%, 7/511), *An. culicifacies* (*s.l.*) (0.8%, 4/511) and *An. aconitus* (0.2%, 1/511), but in very limited numbers. *Anopheles sinensis* was mostly present from June to August with a peak in July but remained present until November (Fig. 4). *Anopheles minimus* (*s.l.*) was less prevalent but displayed a bimodal curve with two peaks in July and September. *Anopheles peditaeniatus* was recorded only from July to October with a maximum plateau in August and September. The survey of mosquito prevalence in cowsheds yielded a slightly different pattern (Fig. 4b). The first difference was the total number of mosquitoes collected in cowsheds, which was almost four-fold higher than in houses, the ratio between cowsheds and houses was 3.77 (1930/511). The same three species were the most prevalent, i.e. *An. sinensis*, *An. minimus* (*s.l.*) and *An. peditaeniatus*, but with different ratios than in human houses. *Anopheles sinensis* was still the most prevalent (48.1%, 929/1930), followed by *An. peditaeniatus* (30.4%, 586/1930) and *An. minimus* (*s.l.*) (18.0%, 347/1930). Three more species were detected in cowsheds at a lower extent: *An. splendidus* (2.4%, 47/1930), *An. culicifacies* (*s.l.*) (0.9%, 17/1930) and *An. argyropus* (0.2%, 4/1930). The main three species were recorded for the same period as in human houses. *An. sinensis* was mostly present from June to September with a peak in July; *An. peditaeniatus* was recorded from July to October, with a peak in August, and *An. minimus* (*s.l.*) displayed the same bimodal curve with peaks in July and September (Fig. 4b). *Anopheles liangshanensis* and *An. maculatus* (*s.l.*) were not found in Man Lve.

### Variation of adult mosquito incidence over time and space in Nong Ling

Adult mosquitoes were collected in the village of Nong Ling (Fig. 3) over the same period and in similar places, i.e. houses (Fig. 4c) and cowsheds (Fig. 4d), as in Man Lve. The overall number of individuals captured in houses (*n* = 479) was in the same range as in Man Lve, with the same three main species, i.e. *An. sinensis* (62.0%, 297/479), *An. peditaeniatus* (23.4%, 112/479), and *An. minimus* (*s.l.*) (12.5%, 60/479). Unlike in houses in Man Lve, *An. peditaeniatus* was the second most prevalent species. The main difference was the population size in cowsheds: 22 in Man Lve and 112 in Nong Ling (Fig. 4). Light traps in cowsheds in Nong Ling yielded a higher number of captured adults (*n* = 1169), although less than in Man Lve. The ratio between cowsheds and houses in Nong Ling was only 2.4 (1169/479). The most prevalent species in cowsheds was *An. peditaeniatus* (48.8%, 571/1169) followed by *An. sinensis* (44.1%, 515/1169) and *An. minimus* (*s.l.*) (6.7%, 78/1169) (Fig. 4d). The bimodal curve of *An. minimus* (*s.l.*) displayed a plateau covering September and October. Beside these three dominant species, four more species were also collected such as *An. culicifacies* (*s.l.*) (1.3%, 6/479), *An. liangshanensis* (0.4%, 2/479), *An. maculatus* (*s.l.*) and *An. splendidus* (0.2%, 1/479) in human houses. *Anopheles aconitus* and *An. argyropus*, rare in Man Lve, were not found in Nong Ling.

### Overall analysis of the prevalence of adult *Anopheles* mosquitoes

**Fig. 5:**
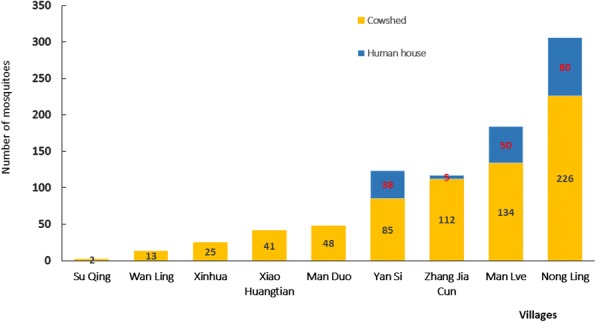
Relative distribution of *Anopheles* mosquitoes in human houses and cowsheds in the nine study villages in October 2015

**Fig. 6:**
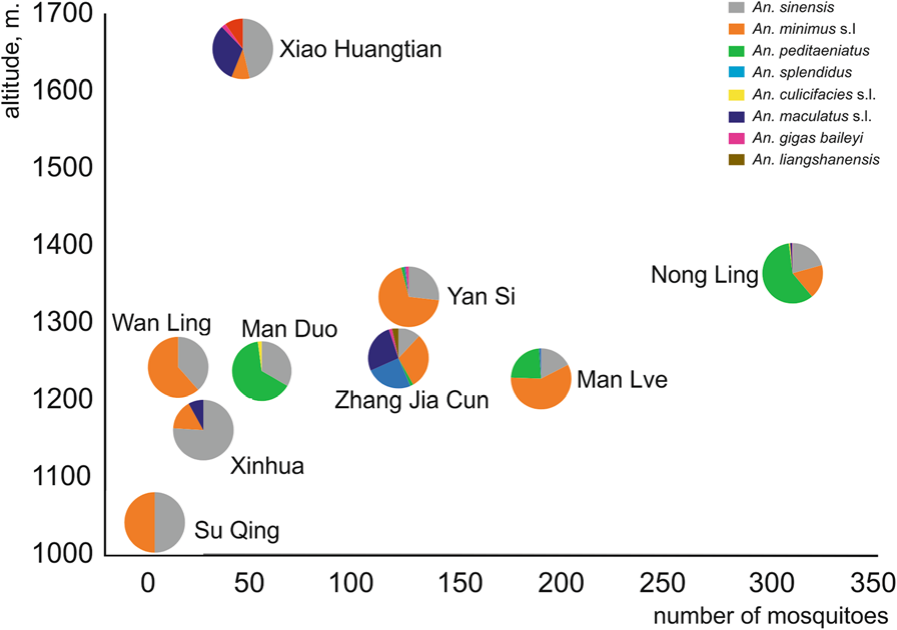
Prevalence of captured *Anopheles* mosquitoes per village according to altitude (m)

When considering the cumulated data in Man Lve and Nong Ling, the most frequent species were *An. sinensis* (50.3%, *n* = 2055), *An. peditaeniatus* (31.6%, *n* = 1291) and *An. minimus* (*s.l.*) (15.8%, *n* = 648). They contributed for 97.7% of the total *Anopheles* mosquitoes collected. *An. sinensis* was the predominant species in both human houses (61.7%, 611/990) and cowsheds (46.6%, 1444/3099). However, *An. minimus* (*s.l.*) was the second largest mosquito species in human houses (22.5%, 223/990). The PCR analysis of the 647 specimens of *An. minimus* (*s.l.*) indicated that 64.8% (419/647) were *An. harrisoni* (former *An. minimus* species C [32]) and 35.2% (228/647) were *An. minimus* (former *An. minimus* species A) (Table 4). One specimen, initially identified as *An. fluviatilis* by morphological identification, was confirmed as *An. harrisoni* by PCR (Table 4). Four specimens of the Maculatus Group were also confirmed as *An. maculatus* by PCR assay (Table 4). The study was extended in October 2015 to seven additional villages in the close vicinity of Man Lve and Nong Ling with the same methods (Fig. 2) to analyze the diversity of *Anopheles* species during a period of higher diversity. The same number of sampling points and same sampling efforts were applied in all the villages. A total of 859 adults were collected during this month over the 9 villages considered. Mosquitoes collected from human houses made up only 20.1% (173/859), while 79.9% were isolated from cowsheds (686/859) (Fig. 5). The number of collected mosquitoes were the highest (> 100 specimens) in four villages: Zhang Jia Cun (*n* = 117); Yan Si (*n* = 123); Man Lve (*n* = 184); and Nong Ling (*n* = 306). The most frequent species in the 9 villages were also *An. sinensis* (23.5%, 202/859), *An. minimus* (*s.l.*) (34.9%, 300/859) and *An. peditaeniatus* (30.2%, 259/859). *Anopheles sinensis* was found in all the sites investigated and *An. minimus* (*s.l.*) in all but one site, Man Duo (Fig. 6). Conversely, *An. peditaeniatus* was found in five out of nine sites only and was highly present in only three sites, Man Lve, Nong Ling and Man Duo (Fig. 6).

**Table 4:** Molecular identification of sibling species

PCR results/Morphological results	*An. minimus* *n* (%)	*An. harrisoni* *n* (%)	*An. maculatus* *n*	Sub-total *n*
*An. minimus* (*s.l.*)	228 (35.2)	419 (64.8)	–	647
*An. fluviatilis*	0	1	–	1
*An. maculatus* (*s.l.*)	–	–	4	4

### Detection of *Plasmodium* spp. in mosquitoes and parous rate

No *Plasmodium* parasite was detected in any captured mosquitoes (Table 5). Out of 295 mosquitoes captured and dissected for parous status, 101 were *An. sinensis* and 194 were *An. minimus* (*s.l.*) With respect to *An. sinensis*, 88 individuals (87.1%) were parous while 180 (92.8%) *An. minimus* (*s.l.*) mosquitoes were also found parous.

**Table 5:** Detection of *Plasmodium* in captured mosquitoes. All test results were negative

*Anopheles* spp.	Total number	Trapped place	
		House	Cowshed
*An. sinensis*	1243	314	929
*An. minimus* (*s.l.*)	510	163	347
*An.peditaeniatus*	608	22	586
*An. sinensis*	812	297	515
*An. minimus* (*s.l.*)	138	60	78
*An. peditaeniatus*	683	112	571
*An. maculatus* (*s.l.*)	4	1	3
*An. liangshanensis*	2	2	0
Total	4000	971	3029

### Blood meal identification

A total of 300 blood samples from trapped mosquitoes were tested. *An. sinensis* was found more zoophilic (27.3%) than *An. minimus* (*s.l.*) (10.7%). *Anopheles minimus* (*s.l.*) displayed more mixed blood meals, either animal/animal (13.1%) or animal/human (14.3%) than *An. sinensis* (3% and 9.1%, respectively) (Table 6). The human blood indices for *An. sinensis* and *An. minimus* (*s.l.*) are 0.21 (28/132) and 0.26 (44/168), respectively.

**Table 6:** Blood meal identification sources in *Anopheles sinensis* and *An. minimus* (*s.l.*)

Species/complex	Blood source, *n* (%)			Mix, *n* (%)		Total
	Pig	Cow	Human	Pig & cow mix	Pig or cow & human mix	
*An. sinensis*	64 (48.5)	36 (27.3)	16 (12.1)	4 (3.0)	12 (9.1)	132
*An. minimus* (*s.l.*)	84 (50.0)	18 (10.7)	20 (11.9)	22 (13.1)	24 (14.3)	168

### Distribution of larvae in potential breeding sites

A total of 407 mosquito larvae were collected in Man Lve and Nong Ling by hand-dipper sampling from four types of habitats such as pond (man-made), pool, ditch and rice field (Table 7). Four *Anopheles* species were identified among the fourth instar larvae and pupae collected (208 specimens in total), i.e. *An. sinensis*, *An. minimus* (*s.l.*), *An. culicifacies* (*s.l.*) and *An. peditaeniatus*. *Anopheles sinensis* (51.0%, 106/208) and *An. minimus* (*s.l.*) (46.2%, 96/208) were the predominant species (Table 7). Ponds (57.2%, 233/407) and rice fields (28.3%, 115/407) were the two preferred breeding sites for the *Anopheles* mosquitoes collected (Table 7).

**Table 7:** Abundance of *Anopheles* taxa found in larval sampling in Man Lve and Nong Ling villages

Site	I-III instar	IV instar and pupa, *n* (%)					Total
		*An. sinensis*	*An. minimus* (*s.l.*)	*An. culicifacies* (*s.l.*)	*An. peditaeniatus*	Sub-total	
Pond	137	64 (66.67)	31 (32.29)	0 (0)	1 (1.04)	96	233
Pool	11	5 (45.46)	4 (36.36)	2 (18.18)	0 (0)	11	22
Ditch	12	1 (4.00)	23 (92.00)	0 (0)	1 (4.00)	25	37
Rice field	39	36 (47.36)	38 (50.00)	1 (1.32)	1 (1.32)	76	115
Total	199	106 (50.96)	96 (46.16)	3 (1.44)	3 (1.44)	208	407

## Discussion

This study is part of the malaria surveillance activities associated to malaria elimination in China, particularly for risk assessment of malaria reintroduction. China has made significant progress on malaria elimination since 2010, and has achieved zero report of indigenous malaria cases within the whole country in 2017 [33, 34]. These achievements were attributed to the promotion of the 1-3-7 approach [35, 36], in which “1-3” are mostly focused on timely case reporting and verification, while “7” is meant to assess the risk of transmission based on entomological information. The latter is considered as an important component of malaria surveillance and response at the elimination and post-elimination stage [37, 38]. The receptivity indicators are thus important for entomological surveillance, in particular in the China-Myanmar border area where a high diversity of *Anopheles* mosquitoes is occurring [37–41].

Tengchong County (TCC) is one of the major malaria endemic counties in Yunnan Province with both *Plasmodium falciparum* and *P. vivax* being transmitted by several *Anopheles* species. The climate and environment are suitable for propagation of malaria vectors and the high cross-border mobility of populations are conditions that favor malaria transmission [5, 6, 12, 14, 18]. However, after several years of malaria control effort, TCC has successfully decreased the incidence of malaria from 35.4/10,000 in 2006 to 2.09/10,000 in 2014 with no indigenous case reported since 2013 [8, 42]. After 2010, the year when malaria elimination program was launched, most malaria cases (40.6%), mainly imported ones, were reported from southern townships within TCC.

Four *Anopheles* species or complexes were previously recorded as predominant malaria vector in TCC, i.e. *An. minimus* (*s.l.*), *An. dirus* (*s.l.*), *An. sinensis* and *An. liangshanensis* (syn. *An. kunmingensis*) [43, 44]. The latter was considered the primary vector of *P. falciparum* malaria with a local transmission in TCC at high altitude (> 1700 m), due to its greater susceptibility to *P. falciparum* compared to *P. vivax* [45]. However, both malaria control interventions (such as LLIN/ITN, IRS) and reduction of rice field surface at high altitude have decreased the density of *An. liangshanensis* populations in line with the number of local *P. falciparum* malaria cases [42, 46]. *Anopheles dirus* was found neither at the adult nor larval stage in this study. This indicates that the population density of *An. dirus* has decreased and might now play a negligible role in malaria transmission. Similar results were reported in neighboring counties [40] and in Hainan Province [47] where *An. dirus* initially present as the primary malaria vector has disappeared [39, 43]. The absence of *An. dirus* might not only be due to vector control activities (particularly the use of LLIN/ITN) but also to the destruction of breeding sites such as forests to develop plantations of cash crops.

In this study, *An. sinensis* was found to be the predominant species in both human houses and cowsheds. This differs significantly from previous reports from neighboring counties [48], where *An. minimus* (*s.l.*) was the predominant taxon. This is particularly important because *An. sinensis* displays specific traits making it a potential threat for malaria elimination. First, *An. sinensis* is associated with rice fields [39] and there is no possibility to eliminate this type of breeding sites. Secondly, *An. sinensis* has been reported as resistant to insecticides such as pyrethroids and Malathion [39, 49]. Thirdly, blood-meal analysis showed that *An. sinensis* displayed a similar tropism to humans as *An. minimus* (*s.l.*). These traits, combined with the predominant abundance of *An. sinensis* in TCC, are major concerns for the success and sustainability of malaria elimination. The exact role of *An. sinensis* in malaria transmission in TCC and, more widely in Yunnan Province, should then be thoroughly investigated. Moreover, regular movements of populations across the China-Myanmar border, owing to the existence of endemic malaria in Myanmar [5, 6], increase the risk of malaria vulnerability in TCC and Yunnan through transmission by *An. sinensis*. This threat must thus be further assessed and modeled while scenarios of risk management must be developed. Furthermore, *An. minimus* (*s.l.*), another malaria vector in TCC, was consistently found in this study with two peaks of density during the year. A large part of the *An. minimus* (*s.l.*) population (64.8%) belonged to *An. harrisoni.* This could explain the lower local malaria transmission in TCC since *An. harrisoni* was reported to be more exophagic and zoophilic than *An. minimus*, its sibling species [40, 50–52]. Furthermore, *An. harrisoni* was also reported to be more adaptable to the environmental changes and flexible in its trophic behavior than *An. minimus* [51, 53–55], which could be a challenge to vector control strategies and entomological surveillance. For instance, the shift of species between *An. minimus* and *An. harrisoni* was reported to be a consequence of vector control measures in Southeast Asia [56]. Comprehensive vector control measures including LLIN/ITN and IRS were indeed conducted in TCC over the last decade [12]. The composition of *An. minimus* (*s.l.*) found in this study may have resulted from vector control measures, as well as environmental changes increasing the proportions of *An. harrisoni versus An. minimus*. Unfortunately, PCR techniques for identification of the *Anopheles* complexes were not used in previous routine surveillance and there is thus a lack of detailed information about the initial distribution of *An. minimus* (*s.l.*) [39]. It is therefore not possible to formally conclude what impacted the current composition of *An. minimus* (*s.l.*) This indicates that more detailed integrative analyses should be conducted to better understand the mechanisms involved in the dynamic of vector populations. A closer attention should also be brought to PCR species identification, an approach to be implemented in routine surveillance at malaria elimination stage and post-elimination stage. Despite the decrease of population density of *An. minimus* (*s.l.*) in TCC, the ecological behavior such as resting or blood-seeking behavior was found to be similar as previously described prior to population decrease [57]. Furthermore, a potential synergistic action of *An. sinensis* and *An. minimus* (*s.l.*) in potentiating malaria transmission should not be ignored. Owing to its zoophilic diet preference and considering the lack of competence for transmission of malaria parasites, *An. peditaeniatus* was not previously reported as a malaria vector [19]. However, the presence of *P. falciparum* in one specimen of *An. peditaeniatus* was recently confirmed by ELISA in Indonesia [58]. Since *An. peditaeniatus* was the second largest population of adult mosquitoes found in this study, further investigation should thus be conducted to monitor the risk of malaria transmission by this species in TCC. Another aspect to consider is the discrepancy between the number of adults and larvae of *An. peditaeniatus* captured. Only three fouth-instar larvae of *An. peditaeniatus* were identified out of 199 *Anopheles* larvae indicating a very low prevalence in all the breeding sites investigated. Conversely, there is a high prevalence of adults in cowsheds. This suggests that the actual breeding sites of this species were most likely missed. It is therefore essential to investigate thoroughly all possible breeding sites for *An. peditaeniatus*.

Environmental factors should also be considered when conducting entomological survey. *An. peditaeniatus* was found in large number in only three localities out of nine and at low level in two more. The two main vectors, *An. sinensis* and *An. minimus* (*s.l.*), were present in all localities and in all but one, respectively. Nevertheless, the main difference is the species richness and the number of individuals between human houses and cowsheds, which might be linked to *Anopheles* blood preference or more favorable living conditions. Although some predominant *Anopheles* species are known as zoophilic, a reduction of livestock may favor malaria re-emergence as some species are quite ubiquitous like *An. sinensis* [59]. Furthermore, models have shown that zoophilic mosquitoes can also play a significant role in the transmission of malaria to humans [60].

Although no *Plasmodium*-infected mosquito was found, the high parous rate of *An. sinensis* (87.1%) and *An. minimus* (*s.l.*) (92.8%) suggests a high daily survival probability [61]. Continuous entomological surveillance and vector control measures are highly recommended even after TCC had officially achieved malaria elimination. Further research should be addressed such as: (i) Investigation of seasonal dynamics of the vectors through the implementation of a weather-based statistical dynamic and climate change model; (ii) Development of a distribution/predictive map of *An. minimus* complex and *An. sinensis* across TCC and the border areas; (iii) Evaluation of the length of the possible transmission season for *P. falciparum* and *P. vivax*; and (iv) Evaluation of the vectorial capacity of *An. minimus*, *An. harrisoni* and *An. sinensis*.

## Conclusions

TCC was granted the malaria elimination certificate by Yunnan provincial authorities in 2016. However, considering the increasing mobility of the local populations, the border location with Myanmar, the positive vector competence of local *Anopheles* populations, and the risk posed by secondary vectors and insecticide resistance, further efforts should be devoted to surveillance, monitoring and development of scenarios for timely response to imported malaria cases. A specific attention should be paid to local environments and variation of vector prevalence when developing scenarios. Large-scale analysis might not be accurate and reliable enough. Precise actions from both local CDC and national program at this border area will be essential for the success of sustainable malaria elimination.
